# Editorial: Adaptation of plants to waterlogging and hypoxia

**DOI:** 10.3389/fpls.2024.1425012

**Published:** 2024-05-21

**Authors:** Najeeb Ullah, Daniel K.Y. Tan, Waqar Ahmad, Silvia Pampana

**Affiliations:** ^1^ Agricultural Research Station, Qatar University, Doha, Qatar; ^2^ The University of Sydney, Plant Breeding Institute, Sydney Institute of Agriculture, School of Life and Environmental Sciences, Faculty of Science, Sydney, NSW, Australia; ^3^ School of Agriculture and Food Sustainability, The University of Queensland, St Lucia, QLD, Australia; ^4^ Department of Agriculture, Food and Environment, University of Pisa, Pisa, Italy

**Keywords:** carbon assimilation, climate change, crop yields, flooding, plant secondary metabolites

Climate change and erratic shifts in weather patterns have become a major issue for global agricultural productivity (Ahmad et al., 2022). For instance, frequent events of extreme weather such as floods and subsequent waterlogging can significantly inhibit the growth and productivity of crops by disrupting oxygen (O_2_) availability to roots. Plant species respond variably to soil waterlogging and hypoxia. While some species may adapt to O_2_-deficient conditions through physiological, biochemical, and molecular modifications, most crops experience substantial growth and yield reductions. In recent years, there has been a significant interest in exploring diverse aspects of plant adaptation to waterlogging and hypoxia. This Research Topic is a collection of coherent research studies that used novel and multidisciplinary approaches to characterize the plant response to waterlogging and hypoxia. A total of 25 papers were submitted of which 14 (2 reviews, and 12 original research studies) were accepted for publication following. These articles cover three main areas: (i) morpho-physiological responses of plants across different developmental phases, (ii) molecular pathways associated with regulation of low oxygen response, and (iii) management technologies for improving crop performance under waterlogged conditions (**Figure 1**).

**Figure 1:**
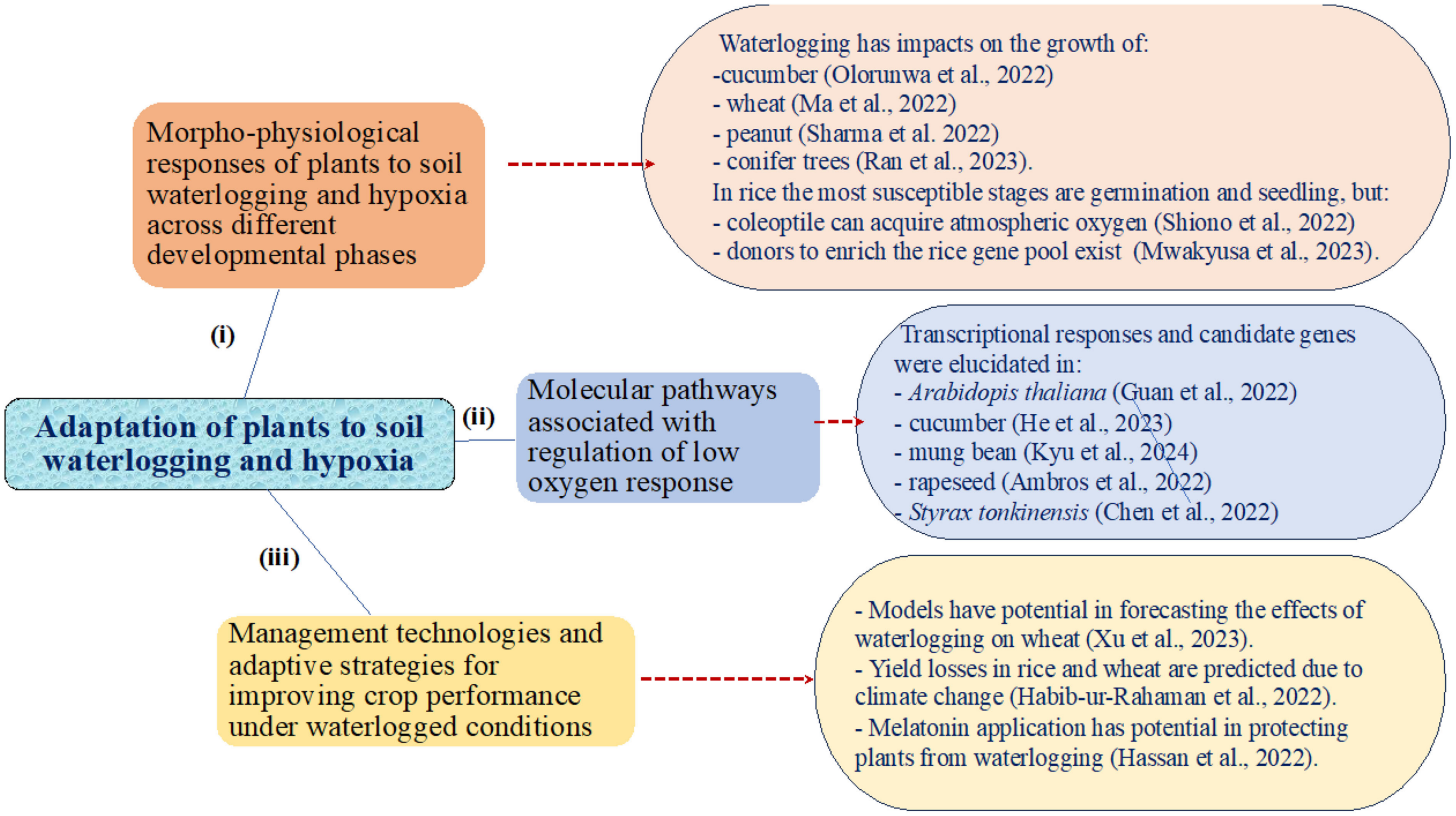
An overview of the main findings of the 14 published papers of the Special Research Topic “*Adaptation of plants to waterlogging and hypoxia*” addressing: (i) morpho-physiological responses of plants across different developmental phases, (ii) molecular pathways associated with regulation of low oxygen response, and (iii) management technologies and adaptive strategies for improving crop performance under waterlogged conditions.

This editorial aims to highlight key advances in the understanding of plant responses to waterlogging and hypoxia, as well as potential strategies for improving crop resilience. Physiological mechanisms governing plant responses to waterlogging have been investigated across varying developmental phases of crops, unraveling the complex regulatory processes associated with growth and yield stability under stressed environments. For example, Olorunwa et al. observed significant inhibition in carbon assimilation in cucumber cultivars exposed to 10-d waterlogging during the early vegetative phase. Similarly, Sharma et al. linked growth inhibition in peanut (*Arachis hypogea* L.) variety DH-86 with impaired photosynthetic pigments, antioxidative enzymes, and chlorophyll fluorescence under waterlogging. Earlier studies also linked poor photosynthesis performance with waterlogging sensitivity in crops such as cotton (Najeeb et al., 2015), and common and durum wheat (Cotrozzi et al., 2021; Ma et al., 2021). Ma et al. quantified the impact of varying waterlogging durations (3, 6 and 9 days) on wheat crops and linked grain yield loss with poor carbon assimilation. Genotypic variations in sustaining photosynthesis and growth under waterlogging (Olorunwa et al.) suggested that developing cultivars with superior PSII photochemistry could improve waterlogging tolerance in crops. Current atmospheric carbon dioxide (CO_2_) levels (in 2024) are off track with the trajectory needed to meet the 1.5°C goal. Elevated CO_2_ levels drive an increase in plant photosynthesis, which may contribute to sustaining photosynthesis under waterlogged conditions as suggested by Najeeb et al. (2017). However, the crops may experience yield losses due to the negative effects of CO_2_ on other physiological processes. The findings emphasize the need for management strategies, including planting time, fertilization, and genotype selection, to mitigate the impact of waterlogging during critical growth phases.

Submergence during germination or just after seeding impacts aerobic metabolisms and limits the growth of most crops. El-Hendawy et al. (2011) suggested that rice genotypes could achieve waterlogging tolerance through rapid water absorption and germination because the coleoptile acted as a snorkel to acquire atmospheric O_2_ to initiate the first leaf elongation and seminal root emergence. Shiono et al. obtained direct evidence for this hypothesis by visualizing the spatiotemporal O_2_ dynamics during submerged germination in rice using a planar O_2_ optode system. Furthermore, using a diverse pool of 200 genotypes primarily landraces and breeding lines, Mwakyusa et al. identified genotypes, which can be used for breeding flooding tolerance rice cultivars. High soil moisture not only herbaceous crops but also trees. Ran et al. analyzed leaf pigment content, root architecture, and respiratory characteristics of three coniferous species and found that species with a higher root proportion in a 40–60 cm soil layer experienced more severe anoxia.

The Research Topic explored the role of endogenous hormones and molecular pathways in understanding the genetic basis of stress tolerance. Chen et al. investigated the molecular responses of *Styrax tonkinensis* under waterlogging stress, focusing on endogenous hormones and phyto-hormone-related pathways. Similarly, the role of exogenous calcium in modulating cucumber response to hypoxia was explored through transcriptome and small RNA analysis (He et al.). The identification of differentially expressed genes related to hormone signaling pathways and antioxidant properties opens avenues for understanding the regulatory mechanisms behind calcium-induced hypoxic stress alleviation.

The impact of climate change on agricultural productivity, especially in food-insecure regions, has prompted research into adaptive strategies. A case study projected 17.2% and 14.1% grain yield losses in rice and wheat crops, respectively, under a changing climate scenario in Pakistan for mid-century (2040–2069) (Habib-ur-Rahman et al.). Moreover, Xu et al. explored the effects of soil temperature and the timing of the waterlogging events on wheat, using two models (i.e., SWAGMAN Destiny and CERES) simulating soil aeration index, a variable used to compute three waterlogging indices. Thus, adoption strategies such as altering sowing times, crop rotation, agroforestry, climate-resilient cultivars, and smart technologies are needed to mitigate the negative effects of climate change.

Hassan et al. reviewed the potential of melatonin for improving plant performance under various abiotic stresses, including those induced by climate change. They suggested melatonin application offers a great potential to protect plants from waterlogging injury through modulating plant physiological mechanisms. Similarly, Ambros et al. studied the transcriptional response of rapeseed cultivars to root zone hypoxia. They found that induction of genes associated with carbohydrate metabolism under short-term (4 and 24 h) hypoxia assisted plants in sustaining carbohydrate status in roots. In addition to management strategies, breeding advancements in genomics have facilitated comprehensive screening for waterlogging tolerance. Genome-wide association studies to identify candidate genes associated with adaptive traits in mung beans revealed five significant associations with five phenotypic traits indicating improved tolerance (Kyu et al.). Similarly, genome-wide analyses on the aldehyde dehydrogenases (ALDH) gene superfamily in the model plant *Arabidopsis thaliana* L. by Guan et al. revealed that 16 AtALDH genes are organized into ten families and distributed irregularly across 5 chromosomes in *Arabidopsis*. These results suggest the integration of molecular approaches, such as marker assisted selection, as promises to accelerate the breeding of climate-resilient crop varieties.

Beyond theoretical insights, the above-described studies bridge the gap between research and practical applications. From evaluating adaptive traits to proposing management strategies, these works contribute valuable knowledge that can be translated into on-the-ground solutions for farmers facing waterlogging challenges. This special topic presents diverse and innovative approaches to mechanistic understanding of adaptation of plants to soil waterlogging and hypoxia (**Figure 1**). These include but are not limited to breeding waterlogging tolerant genotypes and targeting traits such as improved root architecture and modified photosynthetic capability. Similarly, with the introduction of high-throughput phenotyping protocols in crop breeding programs, the identification of genotypes carrying traits linked to waterlogging tolerance can be accelerated. In addition, genetic engineering initiatives aimed at enhancing waterlogging tolerance through molecular analysis, such as transcriptome and proteomic analysis, can identify important genes and pathways involved in low oxygen response.

## Author contributions

NU: Writing – original draft, Writing – review & editing. DT: Writing – review & editing. WA: Writing – review & editing. SP: Writing – review & editing.
